# A Guided Workbook Intervention (WorkPlan) to Support Work-Related Goals Among Cancer Survivors: Protocol of a Feasibility Randomized Controlled Trial

**DOI:** 10.2196/resprot.5300

**Published:** 2016-05-03

**Authors:** Pernille Luxhøj Woods, Lauren Schumacher, Steven S Sadhra, Andrew J Sutton, Anjali Zarkar, Pauline Rolf, Elizabeth A Grunfeld

**Affiliations:** ^1^ Coventry University Centre for Technology Enabled Health Research Coventry United Kingdom; ^2^ University of Birmingham Occupational and Environmental Medicine Institute of Clinical Sciences Birmingham United Kingdom; ^3^ University of Leeds Leeds Institute of Health Sciences Leeds United Kingdom; ^4^ University Hospitals Birmingham National Health Service Foundation Trust Oncology Queen Elizabeth Hospital Birmingham United Kingdom

**Keywords:** oncology, cancer, return to work, intervention, protocol, RCT

## Abstract

**Background:**

Returning to and staying at work following illness is associated with better physical and psychological functioning. Not working has been shown to be associated with reduced self-esteem, lowered self-efficacy, and decreased belief in one's ability to return to the workplace. Although there is a growing body of research looking at what predicts return to work following cancer treatment, there are fewer studies examining interventions targeting return to work.

**Objective:**

The primary objective is to assess the feasibility and acceptability of a theoretically led workbook intervention designed to support cancer patients in returning to work to inform a fully powered randomized controlled trial (RCT).

**Methods:**

This is a multicenter feasibility RCT where the main analysis uses a qualitative approach. Sixty participants (aged 18-65 years) who have received a diagnosis of cancer and who intend to return to work will be randomized to either the WorkPlan intervention group or a usual care group (ratio 1:1). Participants in the intervention group will receive a guided workbook intervention (which contains activities aimed at eliciting thoughts and beliefs, identifying targets and actions, and concrete steps to achieve goals) and will receive telephone support over a 4-week period. The primary outcome measure is time taken to return to work (in days), and secondary outcome measures include mood, quality of life, illness perceptions, and job satisfaction. Data will be collected through postal questionnaires administered immediately postintervention and at 6- and 12-month follow-ups. In addition, interviews will be undertaken immediately postintervention (to explore acceptability of the intervention and materials) and at 12-month follow-up (to explore perceptions of participation in the trial and experiences of returning to work).

**Results:**

Enrollment for the study will be completed in May 2016. Data analysis will commence in April 2017, and the first results are expected to be submitted for publication in late 2017.

**Conclusions:**

Currently no standardized return-to-work intervention based on targeting cancer patient beliefs is in existence. If the intervention is shown to be feasible and acceptable, the results of this study will inform a future full RCT with the potential to provide a valuable and cost-efficient tool in supporting cancer survivors in the return-to-work process.

**Trial Registration:**

International Standard Randomized Controlled Trial Number (ISRCTN): ISRCTN56342476; http://www.isrctn.com/ISRCTN56342476 (Archived by WebCite at http://www.webcitation.org/6gblhEPXd).

## Introduction

### Overview

Returning to and staying in work following illness is associated with better physical and psychological functioning. Not working has been shown to be associated with reduced self-esteem, lowered self-efficacy, and decreased belief in one's ability to return to the workplace [1]. Employment is important not only for individual and societal economic reasons [2] but because being out of work is thought to cause, contribute to, and aggravate adverse health outcomes [3,4]. Furthermore, work is an important component of quality of life [5]. The relationship between unemployment and negative health outcomes is thought to be mediated by factors such as socioeconomic status, financial anxiety, and a stress pathway involving physical changes including hypertension and lowered immunity [6,7]. Although there is a growing body of research looking at what predicts return to work (most commonly defined as returning to work quicker and improved self-reported ability to undertake one's role, or workability) following cancer treatment, there are fewer studies examining interventions targeting return to work.

Over 100,000 people of working age receive a diagnosis of cancer each year in the United Kingdom [8]. Earlier diagnosis and improvements in treatment survival rates have led to an increase in the number of cancer survivors. UK policy reviews have highlighted a need for more research into the challenges of living with cancer [9,10]. For many cancer survivors returning to work is a realistic outcome. Many patients do well following treatment; however, some experience ongoing negative outcomes from the disease or treatment (including pain, fatigue, and low mood) that may impact everyday functioning, including work [11]. Over a quarter of cancer survivors report high symptom burden one year post-diagnosis, even after treatment termination [12]. In addition, many cancer survivors still undergo some form of treatment/monitoring for substantial periods of time following termination of active treatment. Return to work rates of between 23% and 75% have been reported [13], and cancer patients are 1.4 times more likely to be unemployed than healthy individuals [4]. Furthermore, return-to-work rates have been shown to vary across cancer types [14], and longer return to work times have been reported among patients undergoing certain treatments (surgery/chemotherapy) [15], experiencing fatigue [16], or reporting a nonsupportive work environment [17]. Although some cancer types have a high return-to-work rate, we know that across cancer types we see a significant proportion of patients return to work too early or in an inappropriate manner, which results in them taking additional sick leave or leaving the workplace [16]. In addition, a large proportion of cancer patients report modifications in working hours, wages, and work patterns as well as reporting perceived reductions in workability [13]. Cancer survivors have been shown to have similar work-disability levels to those reported in other chronic conditions (eg, stroke, diabetes, heart disease, arthritis) but significantly higher work-disability levels when compared with age-matched adults with no reported chronic condition [18]. This supports the finding that cancer survivors often report difficulties in achieving productivity levels similar to healthy counterparts [19].

Predictors of longer time to return to work include a range of disease and treatment, work-related and psychological factors [20]. The relative role of each of these factors is difficult to determine because few studies directly compare these factors or they focus on either a single cancer type or a mixed-cancer sample. However, a recent study [14] examined these factors across four distinct cancer types and identified that, in addition to optimal symptom management and appropriate workplace adaptations, specific cancer (ie, beliefs about the consequences of cancer) and treatment-related (ie, beliefs about controlling the effects of cancer at work) perceptions predicted return to work.

Cancer patients have reported apprehensions about returning to work related to concerns about ongoing treatments and their level of physical fitness [21]. In addition, depressive symptoms are associated with reduced return-to-work rates, and partial or full resumption of work may help alleviate depressive symptoms by challenging dysfunctional beliefs [22]. Research from noncancer disease groups also supports the importance of psychological factors in the return to work process. Among patients diagnosed with coronary heart disease, depression has been shown to impact functional recovery and predict failure or delay in returning to work [23,24]. Perceptions of illness [25,26,27] and work-related disability (independent of physician report of disability) [28] are also predictive of reemployment and occupational functioning.

In the field of cancer, a number of intervention and trial protocols have been published. Such interventions include a 12-week occupational physician-led intervention focused on increasing physical activity in cancer survivors to support return to work [29]; a case management approach focusing on signposting/referring patients to services (eg, physiotherapy and occupational or psychological therapy) that may support return to work [30]; and a tool that cancer survivors use to guide discussions about working [31]. Although this tool was initially well received, it focused on guiding questions during interactions with employers and healthcare professionals and not on beliefs and barriers that impact workability and work behavior. A recent Cochrane systematic review identified the need for more high-quality randomized controlled trials (RCTs) to enhance return to work among cancer patients [32]. Last, a recent metasynthesis of qualitative research studies highlighted the need for vocational interventions with cancer patients to be person-centred and for such interventions to acknowledge the role of social, clinical, and work-related factors [33].

### Current Study

Feasibility studies are conducted before a main study and are used to estimate key parameters to support the design of a full RCT [34]. This feasibility randomized controlled study will trial and evaluate the WorkPlan guided workbook intervention, a theoretically led intervention aimed at targeting known psychological factors to improve work-related outcomes among cancer survivors. The primary objective of the study is to trial the workbook intervention and data collection materials to ensure that the materials are acceptable to participants and that participants are able to provide full answers. This objective will be met through five aims.

In aim 1, data collection materials will be trialed to ensure that the materials are acceptable to participants. We will identify whether the materials are acceptable to participants and whether participants understand and are able to complete the required tasks.

In aim 2, the recruitment process and feasibility of recruiting participants into the study will be tested. We will observe whether we are able to meet the required monthly recruitment targets, identify which methods of recruitment are most successful in attracting participants into the study, and determine if changes could be made to future studies to improve recruitment.

In aim 3, we will test the acceptability of the randomization process among participants. As part of the final interview process we will discuss the randomization process with participants to determine the level of understanding and satisfaction with the information provided.

In aim 4, we will determine retention in control and intervention groups to the 12-month follow-up. Where possible we will determine reasons for attrition in both arms.

In aim 5, we will conduct the groundwork necessary to obtain data that will be required in the definitive trial to enable a full cost-effectiveness analysis. Measures to be used in a full trial will be administered for acceptability.

The study is registered with the UK Clinical Research Network (UKCRN ID: 19013) and the International Standard Randomized Controlled Trial Number registry [ISRCTN: 56342476]. The protocol version is 4.1, date 11.11.2015. The recruitment status is open (participants are currently being recruited and enrolled into the study).

## Methods

### Eligibility Criteria

Inclusion criteria: patients who have received a diagnosis of breast, gynecological, urological, or bowel cancer that has not been classified as metastatic disease or recurrence; are at least 2 weeks posttreatment initiation; are aged 18 to 65 years; were working at the time of diagnosis; and are not currently working but intend to return to work.

Participants will be recruited to the study from multiple UK hospital sites. We aim to recruit 60 participants who will be randomized into either the intervention or the usual care group. There are currently no clear guidelines for estimating an appropriate sample size for feasibility studies. This is not a hypothesis testing study; the sample size is based on pragmatic assumptions around feasible recruitment figures and the number of participants required to estimate the key parameters around the feasibility of a full RCT.

### WorkPlan Intervention

The WorkPlan package is theoretically led and based around the self-regulation model [35] and goal setting theory [36], which have been applied previously in return-to-work interventions. WorkPlan was developed around an intervention mapping methodology used for designing and implementing complex interventions or programs (interventions that comprise a number of separate elements essential to the functioning of the intervention as a whole). WorkPlan is delivered as a 4-week guided workbook intervention consisting of structured sections and activities to provide guidance and support to patients. The workbook is broken down into 4 chapters that participants are encouraged to work through in turn during each week of the intervention period. The workbook comprises activities aimed at eliciting thoughts/beliefs, identifying targets/actions, and adopting concrete steps to achieve goals. Participants incorporate all elements from the workbook into a personal “return-to-work” plan which they are encouraged to create in the fourth and final week. A resources section is included to signpost participants toward relevant avenues of further support. Multiple copies of the return-to-work planning page will be available to encourage changes to be made when necessary, and these plans can be used as a tool when meeting with employers to aid discussion around returning to work. An intervention manual has also been developed to be used by the researchers during the delivery of the intervention.

#### Intervention Group

Patients in the intervention group will be guided through the initial exercises and given a detailed overview of the workbook. They will be encouraged to discuss the workbook with their partner, family, or friends. Telephone support calls will be made by the researchers at 2 and 4 weeks during the intervention period to discuss progress. The workbook is used during the introductory session, at home during the intervention period, and as a reminder during the return to work process.

#### Usual Care Group

Participants will receive usual care which focuses on clinical care and optimal symptom management and will be offered the workbook at the end of the study. In order to prevent participants from undertaking activities in the workbook, the following precautions have been included in the design: (1) the information sheets and prerandomization discussion do not include the content or focus of the intervention and (2) the workbook will not be made available until the participant’s 12-month follow-up.

Participants in either group may access other information and support relating to work posttreatment but will be asked to record any resources or information they receive or access during the trial.

### Procedure

**Figure 1:**
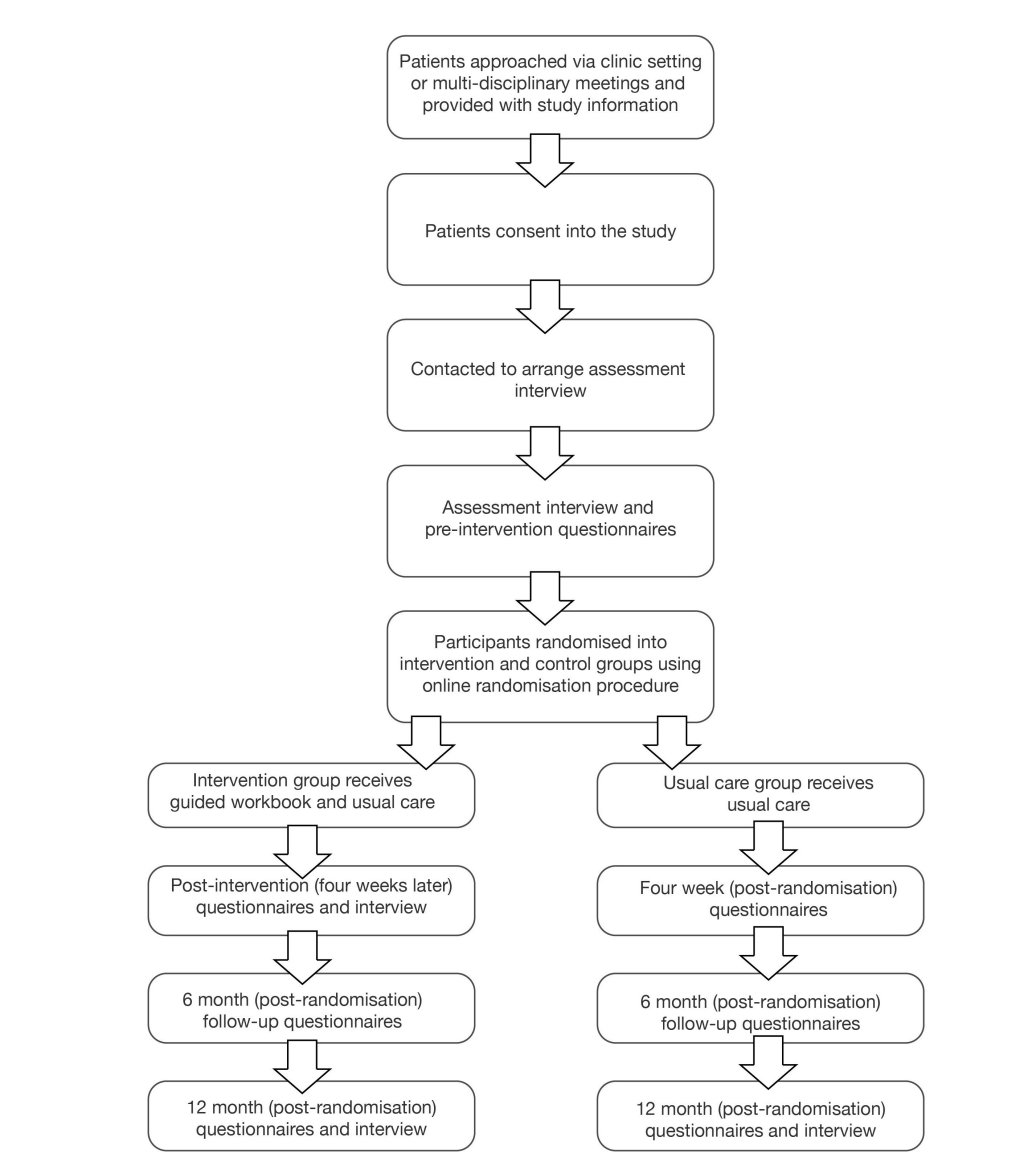
Study flowchart showing allocation to groups.

Participants will be recruited when they are at least 2 weeks posttreatment initiation (Figure 1). Patients will be identified through breast, gynecological, colorectal, or urological cancer clinics; through multidisciplinary team meetings; and by placing posters in clinics, chemotherapy suites, and computerized tomography scan waiting areas. Clinicians will have leaflets and information packs outlining the study and providing contact details available for patients. Study materials have been translated into the five most commonly spoken languages among people of working age in Birmingham (2011 Census): Bengali, Chinese (standard), Polish, Punjabi, and Urdu. Interpreters will be provided if required.

Potential participants will be provided with contact details and asked to contact one of the researchers by telephone or email. Details for the project website will also be displayed on the leaflets and posters, where potential participants can access further information about the study. Patients who express interest in the study will be provided with an information sheet and eligibility screening questionnaire. Eligible participants will be sent an invitation to be interviewed at the hospital or over the telephone; a researcher will outline the study and randomization process, explain the patient information sheet, and obtain written consent (if explained via telephone, researchers will obtain verbal consent after explaining the study and will ask participants to return a written consent form by postal mail). If participants provide additional consent, the researchers will inform their general practitioner about participation in the study. Participants will receive £20 when they complete the assessment interview to cover time and travel expenses.

### Allocation and Stratification

The researchers will randomize participants into one of the two arms using a central online and text system, Sealed Envelope (Sealed Envelope Ltd), at a ratio of 1:1 between the intervention group and usual care group. During the randomization process, participants will be stratified by age (18-50 or 51-65 years) and cancer type (breast, bowel, gynecological, or urological). Patients with different cancer diagnoses may have specific impairments or side effects due to the location of the cancer or the treatments received. Hence, stratifying for cancer type balances out any effects that might be due to this variable. Treatments undertaken during the follow-up period will be monitored in both arms of the trial. Participants are informed about their group allocation (guided intervention or usual care), and participants allocated to the usual care group will be informed that they will be offered the workbook after the 12-month follow-up.

### Blinding

The researchers will be aware of group allocation at randomization and during follow-up in order to provide telephone support to participants in the intervention group. However, the principal investigator will be blind to participant group allocation to reduce bias when analyzing data.

### Data Collection

#### Study Outcomes

The main outcome measures of a full RCT will be used (eg, number of days to return to work and satisfaction with the return-to-work process). At each time point, participants will be asked to recall the date of return to work (paid or unpaid employment, different job, reduced hours/salary, full-time or part-time). Any changes in working status and duties will be documented as will specific reasons for nonreturn to work (eg, unavailability of job, ongoing medical concerns) to determine whether to incorporate specific reasons for nonreturn as measures in a full trial. Secondary outcome measures include mood, satisfaction with return to work, and satisfaction with the return-to-work process. Although not appropriate for a feasibility trial, we would aim to undertake subgroup analysis of the primary outcome measure by cancer type/site in a future definitive RCT.

Data will be collected at 4 time points during the study: baseline, 4 weeks (postintervention), and 6- and 12-month follow-ups. At each time point participants will complete the following questionnaires:

- Illness Perceptions Questionnaire-Revised [37]
- Brief Illness Perception at Work Scale [38]
- Hospital Anxiety and Depression Scale [39]
- Work Ability Index [40]
- Satisfaction with return to work if returned to work (single item)
- Satisfaction with Work Scale [41] (if returned to work)
- EQ-5D-5L (Quality of Life) [42]
- Visual Analogue Scale measure of Quality of Life (single item) [43]

Questionnaire packs will be mailed to participants with a prepaid self-addressed envelope. In addition, participants will be asked to provide details of their use of services and information via text message. A maximum of 4 text messages will be sent to participants at the end of each month for the duration of the study to gather information on their current work status and healthcare utilization. Monthly intervals were chosen because research shows that memory of general practitioner appointments is around 4 weeks, so we could not rely on accurate recall of healthcare utilization at 6-month follow-ups [44,45]. The text-based service can also be used for reminders to participants to complete and return questionnaire packs if there is missing data.

#### Interviews

Twenty participants from the intervention group will be interviewed postintervention and 12 months postrandomization, and 20 participants from the usual care group will be interviewed 12 months postrandomization. Participants will be asked to participate sequentially until the recruitment target is reached. Interviews will be conducted over the telephone or face to face, depending on the participant's preference. The postintervention interview will focus on gaining perceptions of (1) how the intervention was delivered; (2) aspects of the intervention individuals found useful; and (3) compliance with the intervention, how aspects of the intervention were used, and recommendations for change. The 12-month interview will explore (1) experiences of the randomization process, (2) general perceptions of the trial, and (3) the personal return-to-work process of each individual. Members of both groups will be asked about their experiences and how this may have impacted their return to work as well as any additional support received regarding return to work.

#### Data Management

To maintain confidentiality, all participants will be given a unique identifier that will be used on all hard copy and database records. Patient names will not be used. Clinical and research government guidelines will be followed for safe and confidential storage of participant personal data (such as password-protected data files), to which only the research team directly involved in the study will have access. If a participant withdraws from the study, identifiable data which has already been collected with consent would be retained and used in the study, but no further data would be collected from the participant.

### Analysis Plan

#### Qualitative Analysis

Although this is a mixed-methods study, the main focus of the analysis of the study will be qualitative. Interviews will be recorded, transcribed verbatim, and analyzed using the framework method [46] to identify emergent themes.

#### Quantitative Analysis

The purpose of this feasibility study is not hypothesis testing. Furthermore, it is anticipated that the sample size will be underpowered to undertake the full analysis that would be used in a full trial (analysis of covariance adjusting for baseline values). Baseline characteristics will be reported as mean and standard deviations or medians and interquartile ranges for continuous data and as n (%) for categorical data. Differences between the intervention and control groups for the primary outcome measure will be examined. Secondary outcome measures will be assessed using independent samples *t* tests (significance level set at .05).

#### Economic Analysis

Although an economic evaluation is not suitable in the context of a feasibility trial, we will undertake a descriptive economic analysis focusing on the resource usage of the intervention (intervention materials, time, follow-ups/support), self-reported indirect costs including paid sick days/unemployment benefits, and healthcare utilization. The EQ-5D-5L will be used to inform the changes in quality of life over time, and these can contribute to the calculation of quality-adjusted life years in a full economic evaluation.

### Data Monitoring

This is a feasibility trial, so a data monitoring committee will not be convened. However, the project steering committee will review safety and efficacy data throughout the trial. Personal data will be accessed by the research team only and will be stored for 12 months after the study has ended and then moved to a secure archiving facility for 5 years.

### Ethics

Ethical approval for this study has been obtained from West Midlands–Solihull (National Research Ethics Service) Research Ethics Committee (Reference: 15/WM/0166). The principal investigator will communicate any amendments to protocol to members of the research team, who will inform trial participants by postal mail if relevant.

### Harms

Because the trial focuses on a workbook-based intervention aimed at promoting return to work, we do not envisage any adverse events or a need to stop the trial prematurely. It is unlikely that the intervention would cause distress, although participants may experience distress while discussing their work in the context of having experienced cancer. Procedures will be in place for participants to access psychological support services if required.

### Dissemination Policy

Results from the study will be reported and disseminated through publication in peer-reviewed scientific journals and presentations at relevant conferences. A lay summary of the findings of the study will be mailed or emailed to the participants if they express interest.

## Results

The project was funded in March 2015, and enrollment will be completed in May 2016. Data analysis will commence in April 2017, and the first results are expected to be submitted for publication in late 2017.

## Discussion

### Principle Considerations

There is currently no available standardized return-to-work intervention focused on targeting cancer patient beliefs. Previous research [47,48] has demonstrated that both cancer patients and organizations report that such an intervention would be invaluable to facilitate return to work and ensure work retention. Undertaking a feasibility study is critical to inform the planning of a larger, fully powered RCT to improve work-related outcomes among cancer survivors. The results of the study will be used to modify the trial materials and methodology if required and determine likely recruitment and retention rates for a larger trial. If appropriate, the results of the feasibility study will be used to estimate a sample size calculation for a future (appropriately powered) RCT of the intervention with a longer follow-up period. If a fully powered RCT were to demonstrate that the WorkPlan intervention is more effective in supporting return to work than usual care, this would allow us to implement a valuable, cost-efficient tool to support people who have received a diagnosis of cancer in planning and achieving supported return to work as well as greater satisfaction with work and the return-to-work process.

### Methodological Considerations

One strength of this study is that it uses a theoretically based intervention. The study follows the best practice guidelines set out by the Medical Research Council, the UK national funding agency, in the development and evaluation of complex interventions [49] and published recommendations for pilot studies [50-53]. The intervention package was developed in several stages. A review of the literature identified that few studies focusing on return to work had targeted participant beliefs and yet the role of beliefs in the performance of numerous behaviors, including return to work, has previously been documented [54,55]. A prospective questionnaire study was developed and administered to identify which clinical, work-related, and psychological variables influence the return-to-work process among cancer patients. As part of this study, qualitative interviews were undertaken to gain further information about the patients’ vocational aspirations, perceptions of the process of returning to work, and beliefs regarding their ability to return to work. The study demonstrated the role played by illness perceptions and beliefs about the impact of illness on return to work as well as differences in predictive factors across cancer types [14,56,57]. The results of this research were used to map the intervention components through an intervention mapping methodology used for designing and implementing complex interventions or programs. It has been used for over 20 years for systematically designing multifaceted programs involving numerous interventions directed at various individuals and environments [58]. This methodology is suited to the development of a return-to-work program because it is a complex intervention requiring a tailored and multifaceted approach. Further strengths of the study: chosen self-reported outcome measures relate directly to the components addressed through the intervention, resources are available to support a diverse sample within the study, and a qualitative analysis approach will be used. Qualitative methods are increasingly applied in the developmental stages of RCTs of complex interventions [58]. Qualitative methods are often used to evaluate participants’ understanding and experience of an intervention. Individual in-depth interviews allow exploration of why some participants may respond more positively to the intervention and what modifications may be required to suit different groups of participants (eg, differences between cancer types and occupation types, specific gender-based needs).

### Conclusion

This feasibility study may be the first step in the development of an intervention that provides long-term benefits and may have some immediate benefits for the sample who participate. The intervention will provide cancer survivors with the skills and confidence to manage their return to work. The intervention may improve long-term job retention among cancer survivors with the potential to be adapted for other conditions. Furthermore, the intervention may have long-term implications for improving psychological outcomes among cancer survivors through improvements in well-being, mood, and physical functioning, all of which could impact the utilization of national health services.

## Abbreviations

RCT: randomized controlled trial

## Appendix

Multimedia Appendix 2 Approval letter of support from NIHR (RfPB) for the application for funding. Includes committee comments from their review of the application which have all been addressed.
