# Supplementary material for: A Guided Workbook Intervention (WorkPlan) to Support Work-Related Goals Among Cancer Survivors: Protocol of a Feasibility Randomized Controlled Trial
Source: JMIR Res Protoc. 2016 May 3;5(2):e75. doi: 10.2196/resprot.5300 (PMC4890733; doi:10.2196/resprot.5300)
Supplement: Multimedia Appendix 2 [file resprot_v5i2e75_app2.pdf]

NHS
National Institute for Health Research

Central Commissioning Facility
Grange House
15 Church Street
Twickenham
TW1 3NL

Tel: 020 8843 8000
Fax: 020 8843 8001
Email: info@nihr-ccf.org.uk
Web: www.ccf.nihr.ac.uk

Dr Elizabeth Grunfeld
University of Birmingham
Frankland Building (Rm 105)
Edgbaston
Birmingham
B15 2TT

21 March 2014

Dear Dr Grunfeld

Research for Patient Benefit (RfPB) Programme: PB-PG-0613-31088 – A feasibility study of "WorkPlan" - a guided workbook intervention to support work-related goals among cancer survivors

I am writing on behalf of the National Institute for Health Research (NIHR) to let you know the outcome of your application that was submitted to Competition 22 of the RfPB Programme.

I am pleased to inform you that the Committee has recommended that your application be supported. However, the Committee has made the following comments on your proposal some of which will require a satisfactory response from you before we can proceed.

The Committee commended this well designed and presented proposal and recognised the experience of the lead applicant in this field of research. The Committee did, however, identify some areas of concern to which it requested the researchers' response:

- The gender and age ranges in the proposed recruitment strategy (and therefore the potential generalisability of the findings) were questioned by the Committee. It was agreed that the types of cancers to be included placed too much emphasis on recruiting women and that cancers affecting young people had not been included. The researchers were asked to comment on and address this point.
- The researchers are asked to reconsider the exclusion of participants who were not fluent in written and spoken English, which the Committee considered might affect the generalisability of any findings. This is a very important point.
- The definition of "return to work" needs clarification, particularly in respect of phased or partial returns to work and return to unpaid employment. The Committee also agreed that further consideration should be given to the meaning and measurement of satisfaction of returning to work, particularly if a change in work pattern or type is necessary due to the extent of recovery.
- The Committee requested further details on the training that would be available to individuals who would be guiding or delivering the intervention.
- The patient burden was considered to be too onerous and the Committee questioned the need for such a high number of questionnaires.
- Applicants are reminded that any changes to the study design will need to be achieved within the £250,000 funding limit for feasibility studies.

Please respond to the above points as appropriate, confirming your intention to take up this offer of funding. Your letter of response should be emailed to me at the address below no later than 11 April 2014.

Attached to the outcome email are the external peer and public reviewers' comments relating to your application. It has been agreed by the Programme Director that comments from reviewers can often be very helpful to applicants and should be provided to you. It should be noted that the external reviewer comments are always taken in the context of a wider discussion on a proposal and matters such as unduly harsh remarks, seeming misunderstanding or bias, will have been taken into account by the Committee in coming to their decision.

Also find below an overview of the host institution's responsibilities, which contains some important information concerning your project. I would like to take this opportunity to remind you that you should refrain from publicising this funding recommendation until a contract is in place.

Integrated Research Application System (IRAS) Identifier
If your project will require ethical approval and/or any other governance approvals, please provide us with the IRAS identifier for this project in your response to this letter. Please note that you do not need to go through the full process of submitting an ethics request to obtain this; recent changes on the IRAS website http://www.myresearchproject.org.uk/ mean that you are able to obtain an IRAS identifier simply by registering your study. Collecting the identifier at this stage is part of a pilot to try to smooth the permissions and approvals pathway for researchers as part of the Health Research Authority's work.

Contracting process
Once the Committee has approved your responses, the finances and intellectual property rights (IPR) for your project will be reviewed by the NIHR Central Commissioning Facility and you will be asked to provide further information on these items before a contract for your project can be issued.

A draft version of the standard contract can be found on our website by following this link http://www.ccf.nihr.ac.uk/RfPB/Documents/NHS%20Trust%20Contract.pdf. Please note that the terms and conditions of this contract are subject to review and amendment at the discretion of the Department of Health. By submitting an application to the Programme you have agreed to be bound by the standard terms and conditions of the contract as stated in the 'Guidance to Applicants'. In order to prepare for the contractual negotiations, I recommend that you read and review the contract in full and in particular the IPR and Warranties and Liabilities terms and conditions. In light of these conditions, please clarify with local R&D management whether there are any third party rights in the background IP which may affect the research, as these will need to be stated clearly in schedule C of the contract. Please also ensure that your plans for managing the foreground IP are in line with the local IPR policy of the host organisation. As described in condition 15.2, our preferred position is that the foreground IP of this research project shall vest in the contractor, given that an NHS body or other provider of NHS services is best placed to realise any patient benefits flowing from the research. We will request clarification on all of these issues during the contractual negotiations.

NIHR Clinical Research Networks (CRN)
If your study involves the NHS or NHS patients we expect you to apply, where appropriate, for consideration for NIHR CRN support in England and/or its equivalents in the devolved administrations and subsequent inclusion in the NIHR CRN Portfolio Database. Please notify the relevant network of this provisional offer of funding as soon as possible. If your study is deemed eligible for consideration for NIHR CRN support, we expect you to:

- keep your study record on the Portfolio Database up to date
- upload your recruitment data onto the Portfolio Database on a monthly basis. Please note that the NIHR CRN will share this data with us through the production of quarterly reports.

Information on the NIHR CRN support available to researchers can be found at http://www.crncc.nihr.ac.uk/Resources/NIHR%20CRN%20CC/Documents/Brochures/CRN%20core%20offer_Nov2012_A4.pdf and information about gaining NHS permission for clinical research and using the NIHR Coordinated System for gaining NHS Permission (CSP) can be found at http://www.crncc.nihr.ac.uk/Resources/NIHR%20CRN%20CC/CSP/Documents/CSP_May2012_DL_v

s4_FINAL.pdf. To access these leaflets, please copy and paste the above links into your internet browser.

NIHR Research Design Service (RDS)
In order to continue to build collaborations between the RfPB Programme and the RDS, you are encouraged to share the Committee's feedback with the RDS advisors who considered this proposal, if applicable.

If you have any questions regarding the decision of the Committee, or any matters relating to the RfPB Programme, please do not hesitate to contact me.

Yours sincerely

Bob Scott
Email: bob.scott@nihr-ccf.org.uk
Telephone: 020 8843 8043

National Institute for Health Research

Overview of the grant holders' project monitoring responsibilities

The contractual responsibilities of the grant holders are described in the standard Department of Health (DH) research contract. It is recommended that you familiarise yourself with all the terms and conditions of the contract and ensure that these are acted upon during the course of the research. This document contains the main actions that the chief investigator will need to fulfil in order to satisfy DH's project monitoring requirements.

1. Submission of favourable ethical opinion
For research funded through the National Institute for Health Research (NIHR), DH requires, where appropriate, evidence that a favourable opinion has been granted by a Research Ethics Committee. Please email copies of any relevant documentation to the Research for Patient Benefit (RfPB) Programme Manager (PM) quoting your project's NIHR reference number. By default, NIHR Central Commissioning Facility (CCF) will neither allow projects to start their research nor initiate payments until this evidence has been provided.

2. Submission of the start certificate
In order to ensure that payments for research projects are not made in advance of need, RfPB makes use of a formal start certificate to record the actual start date. Once the contract is signed and evidence of a favourable ethical opinion has been received, please keep the PM informed of the anticipated start date for the project. You will need to send an email confirming the date once the research activities are underway. The PM will then provide you with a start certificate, which will need to be signed by an authorised signatory of the NHS organisation administering the award, and returned to the CCF offices.
DH wishes to see a dramatic and sustained improvement in the initiation of clinical research. In the event that the actual start date is more than three months after the contracted start date, the host organisation will be required to sign a variation to contract that will be issued by the PM.
Please be aware that to avert serious delays a drop dead date (six months after the estimated start date) has been included within the contract.

3. Completion of progress reports and financial statements
During the course of the project, the grant holders are required to provide verbal or written reports of the technical progress on the project and details of the actual expenditure on the research activities. Template progress report forms will be made available either by email or through CCF's online research management system (RMS) https://ccfrms.nihr.ac.uk/Login. The chief investigator should convey an overview of progress made since any earlier report. The report is designed to capture any material changes to the research or staffing, a summary of progress to date, progress against the project milestones, a list of presentations and publications, background and foreground intellectual property (IP) positions and matters requiring further attention by the PM.
Typically, the first interim report is expected 12 months after the actual start date for your project and subsequent reports are required at 12 month intervals. Where a project is contracted to run for 12 months or less, an interim report is required after six months. It is worth factoring the interim report due dates into your research plan.
Template annual statements of expenditure will be issued at the start of each new financial year. These will need to be completed by the finance officer at the host organisation and should capture all the expenditure incurred by the host and partner organisations to deliver the research during the course of the preceding year. Please note that the host organisation is required to maintain proper financial records relating to the research at all times during the research period and for six years after the project's end. DH reserves the right to request further financial information about projects funded through the NIHR programmes at any time.

4. Variations to contract
If at any time it appears likely that any provision of the contract, in particular the research, needs to be varied, please notify the PM as soon as possible about the nature and rationale for the anticipated change. The PM may ask you to complete a variation to contract request form (or an extension request form) so that the proposed change can be considered by one of the programme's authorities. If the change is approved, the PM will issue a formal variation to contract to capture the change. This document will need to be signed by an authorised signatory at the host organisation and countersigned by DH.

5. Exploitation of IP
Grant holders are expected to be aware of the terms and conditions in relation to the IP rights of the research contract. You should work with the host organisation to identify, protect and maintain IP in accordance with their standard institutional IP policy. In particular, you are required to inform the PM of any results which are capable of being exploited either by direct adoption into the healthcare service or via commercialisation. The host organisation will also need to seek prior written approval from CCF if it wishes to use a third party (excluding professional advisors) to carry out exploitation activities with respect to the foreground IP funded through this award. CCF is interested in following up on the impact of research funding, so further information on directly exploitable IP arising from your project may be requested once the project is complete.

6. Completion of a final report
A draft final report on the research will need to be submitted within 14 calendar days of the contracted end date for the project. A template final progress report form will be provided by the PM in the last month of the project together with a form to provide the final statement of expenditure. Within the final progress report you will be expected to give a comprehensive overview of the work undertaken, including a lay/plain English summary, summary of the research findings (including background, aims and objectives, methodology, key findings, expected impact and conclusions), outline any variations from the original proposal, next steps to patient benefit and the dissemination strategy. RfPB is particularly interested to learn about the experiences of research teams stemming from the patient and public involvement (PPI) in their projects, so further information on PPI in your project may be requested once the project is complete. The final payment will not be made on a project until a satisfactory final report and financial statement has been received.
